# Alexithymia, dissociation and emotional regulation in eating disorders: Evidence of improvement through specialized inpatient treatment

**DOI:** 10.1002/cpp.2665

**Published:** 2021-09-02

**Authors:** Paolo Meneguzzo, Alice Garolla, Elisa Bonello, Patrizia Todisco

**Affiliations:** ^1^ Eating Disorders Unit Casa di Cura “Villa Margherita” Arcugnano Italy; ^2^ Department of Neuroscience University of Padova Padova Italy

**Keywords:** alexithymia, anorexia nervosa, binge eating disorder, bulimia nervosa, dissociation, emotional regulation

## Abstract

The research into emotional regulation in eating disorders (EDs) has shown specific impairments and maladaptive coping strategies in patients, and there is an increasing interest in the role of the emotional domain in the treatment outcome. This study aims to evaluate the effect of a specialized inpatient treatment characterized by both an intensive and comprehensive standardized multidisciplinary programme based on cognitive–behavioural therapy and a flexible and personalized component implemented by third‐wave interventions. A cohort of 67 female ED patients (anorexia nervosa = 28, bulimia nervosa = 28 and binge eating disorder = 11) underwent an evaluation of emotional regulation difficulties, alexithymia and dissociative symptomatology at admission to a specialized ED ward. The psychological modifications were subsequently re‐evaluated upon discharge, after an inpatients treatment of 60 days, examining specific changes in the specific psychopathology. A significant improvement after specialized ED treatment was shown in alexithymia, emotional regulation difficulties and dissociation symptoms, with higher effect sizes in patients with higher alexithymia scores. As regards the specific effect of the psychological improvement, changes into alexithymia scores have shown specific correlations with ED psychopathology (*p* < 0.010) and with difficulties in emotional regulation (*p* < 0.010) in patients with higher alexithymia levels at admission. Emotional regulation and dissociation should therefore be evaluated in ED patients and may be improved with specific therapeutic approaches, while alexithymia remains a clinical trait, even with a significant reduction.

**Abbreviations**
AN: anorexia nervosa
BED: binge eating disorder
BN: bulimia nervosa
DERS: Difficulties in Emotion Regulation Scale
DES‐II: dissociative experience scale
ED: eating disorder
EDE‐Q: eating disorder examination questionnaire
GSI: global severity index
SCL‐90 R: Symptom Checklist‐90‐Revised
TAS‐20: Toronto Alexithymia Scale

**Key Practitioner Message**

- Alexithymia and dissociation should be taken into consideration in eating disorders assessment and treatment.
- Inpatient eating disorder treatment could improve alexithymia, dissociation and emotional dysregulation.
- The results suggest a specific linkage between alexithymia and eating psychopathology.

## 1 BACKGROUND

According to the transdiagnostic approach to eating disorders (EDs), in addition to the specific psychopathology resulting from abnormal eating habits (C.G. Fairburn et al., 2003; M. Solmi, Gallicchio, et al., 2018), researchers identified a more comprehensive psychopathological core characterized by affective comorbidities, interpersonal and interoceptive difficulties, ineffectiveness and a drive for thinness (M. Solmi, Collantoni, et al., 2018). Based on the transdiagnostic perspective, various treatment protocols focusing on dysfunctional schemas and behaviours have been proposed (C.G. Fairburn et al., 2003; Murray et al., 2019), but treatment outcomes remain unclear (J. Linardon, Wade, et al., 2017). Dissociation, alexithymia and emotional dysregulation have been identified as relevant comorbidities that could interfere with eating‐disordered patients' improvement and recovery, but in the current literature, there is no consensus as for effective ways to reduce these symptoms (Fernández‐Aranda et al., 2008; Gramaglia et al., 2020; Thompson‐Brenner et al., 2019; Westwood et al., 2017). Indeed, even though outpatient psychotherapy is recommended as first‐line treatment, the recovery rates are still inadequate, and residential programmes are needed for patients who have severe symptoms or comorbidities that cannot be treated through less intensive approaches; however, there is not much data available on the benefits of inpatient treatment (Thompson‐Brenner et al., 2019).

Emotional regulation, which is defined as “the ability to identify and modulate the experience and expression of emotions,” has recently been proposed as a potential transdiagnostic therapeutic target for ED patients (Mallorquí‐Bagué et al., 2018) due to the increasing evidence that ED patients have difficulty identifying and modulating emotions. Patients present dysfunctional, maladaptive psychological features such as suppression, rumination or emotional avoidance to manage emotions (Aloi et al., 2017; Mallorquí‐Bagué et al., 2018; Meneguzzo et al., 2020), and the eating disorder symptoms were used as a coping strategy for unbearable emotions (Moulton et al., 2015; K. Trottier et al., 2016; K. Trottier & MacDonald, 2017). Indeed, early data suggest there may be benefits in treating EDs by implementing an emotional regulation protocol (Thompson‐Brenner et al., 2019); however, the results are still preliminary and show conflicting evidence (Caslini et al., 2015).

The inability of ED patients to recognize and report emotions has been correlated with alexithymia and dissociation, two dysfunctional psychological features that patients use to cope with intolerable or frightening emotions: unrecognizable feelings, or disintegrate consciousness, memory and identity (Grabe et al., 2000; Nowakowski et al., 2013). Numerous studies conducted on various clinical populations have confirmed the association between these two psychological features by evaluating the co‐presence of maladaptive defence strategies in response to stressful events (Cascino et al., 2020; Grabe et al., 2000). However, few studies in the ED field have evaluated whether there is a connection between alexithymia, dissociation and psychopathology (De Berardis et al., 2009; Franzoni et al., 2013), and the results are considered preliminary (Longo et al., 2020).

Indeed, not enough is known about the coexistence of emotional dysregulation, dissociation and alexithymia in ED patients nor the possible effects of interventions proposed for treating these constructs. Thus, this study aims to evaluate whether general and specific psychopathology is worsened by the presence of alexithymia, as the current literature suggests (Gramaglia et al., 2020), and to determine if a multidisciplinary inpatient treatment for EDs can reduce alexithymia and dissociation. Finally, we aim to point out possible relationships among specific and general psychopathological target variables.

## 2 METHODS

### 2.1 Clinical sample

A cohort of consecutive patients admitted to the ED inpatient treatment ward of the *Casa di Cura Villa Margherita* in Arcugnano (Vicenza), Italy, were enrolled in the study between March and October 2019. The inclusion criteria were (1) any ED diagnosis according to DSM‐5 criteria; (2) between 14 and 60 years of age; (3) no severe psychiatric (i.e., psychotic symptoms, bipolar disorder and severe personality disorders) or medical comorbidities, neurological trauma or disorder, or drug addiction.

An informed consent form was signed by each participant (and their parents if patients were below 18 years old). The study was approved as part of the clinical evaluation of ED patients hospitalized in the *Eating Disorders Unit* by internal review committee at the *Casa di Cura Villa Margherita* and from the Vicenza Ethical Committee (47/21), and it complies with the provisions of the Declaration of Helsinki.

### 2.2 Treatment protocol

The treatment protocol was characterized by both individual and group psychological intervention focused on the psychopathological core of ED and comorbidities. The treatment used a Cognitive Behavioural Therapy (CBT) protocol implemented with psychological techniques focusing on specific needs emerged in the course of the examination of the patient's history (e.g., eye movement desensitization and reprocessing [EMDR] and mindfulness) and was run alongside nutritional rehabilitation (see Todisco et al., 2020, for details). The duration of the treatment was 60 days, in accordance with the National Health System legislation, and patients were admitted to the facility only after at least one outpatient treatment failed.

### 2.3 Material

The patients were evaluated at the beginning of their inpatient treatment (the first week after their admission) and at discharge (the week before the treatment ended) using several self‐reported questionnaires: (1) the Symptom Checklist‐90‐Revised (SCL‐90R) for general psychiatric symptoms and psychological distress (Derogatis & Lazarus, 1994), Cronbach's α = .977. In accordance with the recommendations outlined by the previous literature, two specific subscales were considered—depression (DEP) and anxiety (ANX), as well as the global score. The cut‐offs of the subscales are Global Severity Index (GSI) = 0.60, DEP = 0.73, ANX = 0.75 (Schmitz & Hartkamp, 2000); (2) the Eating Disorder Examination Questionnaire (EDE‐Q) for the assessment of specific eating disorder psychopathology (C. Fairburn & Beglin, 1994), with a cut‐off for the total score of 2.50 (Rø et al., 2015); Cronbach's α = .826; (3) the Difficulties in Emotion Regulation Scale (DERS) for the evaluation of emotion regulation (Gratz & Roemer, 2004), with higher scores indicating greater difficulties with emotions; Cronbach's α = .811; (4) the Dissociative Experiences Scale – II (DES‐II) for the evaluation of dissociative experiences (Schimmenti & Caretti, 2016), the cut‐off for high level of dissociation is 30 (Carlson & Putnam, 1993); Cronbach's α = .939; (5) the Toronto Alexithymia Scale–20 (TAS‐20) for the measure of alexithymia, with a cut‐off score of 61 for alexithymia (Leising et al., 2009; Taylor et al., 1996), Cronbach's α = .780.

### 2.4 Statistical analysis

Baseline differences among patients belonging to diverse diagnostic categories were evaluated with ANOVA and confirmed with an ANCOVA analysis with BMI as a covariate. The sample was then considered as an ED transdiagnostic sample due to the psychopathological similarity. In accordance with data from the literature, we used the TAS‐20 cut‐off (61 points) to create two subgroups—with and without alexithymia symptomatology—and analyses of the alexithymia subsamples were performed using *t* tests for independent samples. Demographic differences between completers and non‐completers were evaluated with *t* tests for independent samples. We conducted a chi‐square analysis to evaluate the distribution of the patients with and without alexithymia in the completers and non‐completers subsamples. The evaluation of the changes after the treatment was done with paired *t* tests. We analysed correlations (Pearson's *r*) to examine correlations between the delta (post‐ minus pre‐treatment) questionnaires' scores. Effect sizes were evaluated using Cohen's *d* (small <0.5, medium >0.5 and <0.8, large >0.8). The data were analysed using the IBM SPSS Statistics 25.0 software (SPSS, Chicago, IL, USA). To control the multi‐comparison bias, we used the Bonferroni correction, and only *p* values ≤0.01 were considered significant.

## 3 RESULTS

### 3.1 Baseline evaluation

The included sample was composed of 100 female patients with anorexia nervosa (*n* = 53), bulimia nervosa (*n* = 36) and binge eating disorder (*n* = 11). The mean age was 28.27 ± 12.07 years (14–60 years), and the mean BMI was 20.76 ± 9.55 kg/m^2^ (11.17–64.78 kg/m^2^). The psychological evaluation of the sample is reported in Table 1. No differences were found between the samples, even when running an ANCOVA analysis with BMI as a covariate.

**TABLE 1:** Psychopathological description of the sample

	AN *n* = 53	BN *n* = 36	BED *n* = 11	F (*p)*
SCL‐90R GSI	1.69 (0.77)	1.81 (0.79)	1.49 (0.64)	0.722 (0.489)

EDE‐Q global score	3.61 (1.71)	4.08 (1.25)	2.97 (1.09)	2.399 (0.096)

DERS tot	53.09 (56.3)	43.56 (54.14)	29.20 (50.33)	0.919 (0.402)

DES‐II tot	16.96 (15.15)	17.38 (12.10)	12.61 (6.86)	0.523 (0.595)

TAS‐20 tot	71.42 (11.61)	63.43 (11.32)	63.00 (4.90)	6.521 (0.020)


*Note*: Means and standard deviations in parentheses.

To measure the differences linked to alexithymia, the patients were divided into two subgroups based on a cut‐off score of 61 on the TAS‐20 (A+ for scores >61, A− for scores <61; Taylor et al., 1996). The A+ subgroup (*n* = 66) showed significantly higher specific ED psychopathological scores than the other subgroup based on the EDE‐Q global score (A+ = 4.01 ± 1.44, A− = 3.13 ± 1.55, *t* = 2.77, *p* = 0.007, *d* = 0.588), but no differences were found in general psychopathology scores based on the SCL‐90‐R total score (A+ = 149.65 ± 73.33, A− = 125.15 ± 85.75, *t* = 1.480, *p* = 0.142, *d* = .307). For emotional regulation, patients with alexithymia showed a higher DERS total score (A+ = 125.70 ± 26.08, A− = 97.39 ± 29.88, *t* = 4.846, *p* < 0.001, *d* = 1.01), but no differences were found regarding dissociative symptoms (A+ = 18.02 ± 13.36, A− = 13.57 ± 12.77, *t* = 1.483, *p* = 0.142, *d* = .341).

### 3.2 Evaluation at the end of the inpatient treatment

The treatment protocol was completed by 67 patients (A+ = 48 [73%], A− = 19 [58%]) who participated in the evaluation prior to discharge. At baseline, no differences emerged in regard to psychopathological scores between completers and non‐completers in the two alexithymia subgroups. Regarding the diagnosis, non‐completer patients were anorexia nervosa patients (25 out of 53 [47%]) and bulimia nervosa patients (8 out of 36 [22%]). Thus, the completers sample was composed by 28 AN patients, 28 BN patients and 11 BED patients. The completers demographic characteristics were mean age 28.69 ± 12.34 years and mean BMI 21.07 ± 10.09 kg/m^2^. No significant demographic differences were found as regard age and BMI between completers and non‐completers. The differences in distribution of A+ and A− patients in the completers and non‐completers subgroups (completers: A+ = 48, A− = 19; no‐completers: A+ = 19, A− = 14; χ2 (1) = 1.979, *p* = 0.160) did not turn out to be significant.

Specialized multidisciplinary treatment proved to be effective in reducing specific psychopathology and dissociation (see Table 2 for pre‐and post‐test analysis).

**TABLE 2:** Pre‐post analyses for both alexithymia sub‐samples.

	A− (*n* = 19)				A+ (*n* = 48)			
SCL‐90R	Pre	Post	*t* (*p*)	*d*	Pre	Post	*t* (*p*)	*d*
GSI	1.52 (0.69)	1.16 (0.62)	2.582 (0.019)	0.549	1.71 (0.72)	1.24 (0.70)	4.487 (**<0.001**)	0.662
DEP	2.14 (0.70)	1.61 (0.81)	1.956 (0.086)	0.700	2.31 (0.99)	1.61 (0.81)	3.777 (**0.001**)	0.774
ANX	1.84 (1.10)	1.27 (0.86)	2.312 (0.050)	0.577	1.88 (1.09)	1.28 (0.95)	3.342 (**0.003**)	0.587
EDE‐Q global score	2.90 (1.36)	2.00 (0.96)	2.518 (**0.010**)	0.739	3.93 (1.43)	2.79 (1.47)	5.858 (**<0.001**)	0.786
DERS total	93.74 (26.30)	64.58 (48.22)	2.919 (**0.009**)	0.751	124.81 (27.53)	71.81 (56.10)	6.538 (**<0.001**)	1.199
DES‐II total score	12.70 (11.20)	10.10 (7.15)	0.991 (0.360)	0.277	20.23 (14.74)	13.91 (15.44)	1.978 (0.065)	0.419
TAS‐20 total score	55.07 (5.32)	51.07 (7.41)	1.247 (0.233)	0.631	74.77 (7.41)	67.74 (9.18)	5.665 (**<0.001**)	0.843

*Note*: Means and standard deviations in brackets. Significant differences were reported with bold characters.

Abbreviations: ANX, anxiety; A+/−, patient subgroups with/without alexithymia symptomatology according to the cut‐off score of 61 points; *d*, Cohen's *d*; DEP, depression; DERS, Difficulties in Emotion Regulation Scale; DES‐II, dissociative experience scale; GSI, global severity index; EDE‐Q, eating disorder examination questionnaire; SCL‐90R, Symptom Checklist‐90‐Revised; TAS‐20, Toronto Alexithymia Scale.

### 3.3 Correlation analyses

Upon examining the relationships among the differences between discharge and admission of the psychopathological questionnaires of the completers, we found different correlations between variables in the two alexithymia subgroups. Only A+ patients showed correlations between the TAS‐20, SCL‐90‐R, the EDE‐Q and the DERS (see Table 3 for details).

**TABLE 3:** Correlations in completers among the differences of the psychopathological variables due to the inpatient treatment (discharge – Admission).

	SCL‐90R			EDE‐Q global score	DERS total	DES II total score	TAS total score
	GSI	DEP	ANX				
A−							
SCL‐90R GSI	‐						
SCL‐90R DEP	.869 ^**^	‐					
SCL‐90R ANX	.714 ^**^	.544 ^**^	‐				
EDE‐Q global score	−.218	−.200	.227	‐			
DERS total	−.038	−.338	−.700	.227	‐		
DES II total score	−.216	.001	.188	−.700	−.107	‐	
TAS total score	−.394	.633	−.393	−.393	−.492	−.306	‐
A+							
SCL‐90R GSI	‐						
SCL‐90R DEP	.872 ^**^	‐					
SCL‐90R ANX	.839 ^**^	.691 ^**^	‐				
EDE‐Q global score	−.045	.094	−.038	‐			
DERS total	−.299 ^**^	−.161	−.216	.174	‐		
DES‐II total score	.444	.367	.312	.282	−.167	‐	
TAS‐20 total score	.159	.189	.088	.550 ^**^	.491 ^**^	.395	‐

Abbreviations: ANX, anxiety; A+/−, patient subgroups with/without alexithymia symptomatology according to the cut‐off score of 61 points; DEP, depression; DERS, Difficulties in Emotion Regulation Scale; DES‐II, dissociative experience scale; EDE‐Q, eating disorder examination questionnaire; GSI, global severity index; SCL‐90R, Symptom Checklist‐90‐Revised; TAS‐20, Toronto Alexithymia Scale;

** *p* < 0.01.

## 4 DISCUSSION

Maladaptive personality traits and mental processes, such as alexithymia and dissociation, have been identified as a possible target of ED treatments due to their importance in emotional regulation deficits. In this study, we aim to evaluate (1) the differences in the clinical and psychological presentation of ED patients with and without alexithymia and (2) the effect of a specialized multidisciplinary treatment protocol in reducing these symptoms.

As proved by previous literature, the psychological evaluation at the time of admission to the ward showed that ED patients with alexithymia are characterized by a worse specific ED psychopathology than those without alexithymia (Nowakowski et al., 2013). We only partially confirmed our first hypothesis because we did not find any differences in general psychological symptoms between ED patients with and without alexithymia. This could be due to the severity of the sample included in our study—the patients who were admitted to the residential programme had high levels of depression or anxiety. Their scores at admission were different from those of outpatients or general populations included in previous studies (Hemming et al., 2019; Lenzo et al., 2020). Alexithymia has shown to be a stable trait in ED patients, even after the reduction of depression and anxiety (Nowakowski et al., 2013), and has been pointed to be the possible explanation of the absence of emotional responses to social exclusion in AN patients (Meneguzzo et al., 2020). A study with a 3 years follow‐up has found that alexithymia have a negative prognostic effect into outcome (Speranza et al., 2007) but data are still preliminary and specific interventions into emotional and feeling recognitions could have a positive effect into psychopathological profile of the patients. Moreover, we found higher degrees of difficulty in emotional regulation, showing a connection between alexithymia and emotional dysregulation that previous literature has already shown in different psychiatric conditions and that have a negative impact in the psychopathology (Torrado et al., 2018). From this prospective, eating could be a dysfunctional coping strategies for emotional dysregulation, and alexithymia could amplified these responses due to the difficulties to understand the own feelings and mental states (Spence & Courbasson, 2012).

The most interesting data from our analysis concern the effect of ED specialized treatment. Indeed, our results provide evidence that a specific treatment approach may help to improve emotional regulation, alexithymia and dissociation. A recent review of the literature has pointed out the absence of longitudinal data about the modification of alexithymia in ED patients (Westwood et al., 2017), and our study could provide the first step in understanding the possible changes of this specific psychological construct after implementing psychotherapeutic approaches in severe patients. Moreover, our data are in line with recent literature, which suggests that using flexible interventions based on the history of patients that includes specific psychological techniques may improve maladaptive schema or behavioural responses (J. Linardon, Fairburn, et al., 2017; Todisco et al., 2020).

When we looked at the effects of the inpatient treatment, we found larger effect sizes in the reduction of DERS and TAS‐20 in patients with higher scores of alexithymia compared with the lower scores subgroup. However, the TAS‐20 scores remain clinically significant for alexithymia even after the inpatient treatment in A+ patients, data that are corroborated by the literature data that considered this feature as a personality trait (Nowakowski et al., 2013). The changes in the TAS‐20 scores are also correlated with a reduction in EDE‐Q scores. These results show that there is a linkage between eating psychopathology and alexithymia, and they are in line with previous results that showed a connection between AN outcome and alexithymia (Gramaglia et al., 2020), suggesting that this aspect should be studied across the whole spectrum of EDs. The emotional dysregulation reported and the difficulties in the management of their emotional states are in line with the interpersonal model of EDs (Treasure et al., 2012), and they reinforce the idea that comorbidities in ED patients who receive residential treatment should be systematically evaluated and taken into consideration in the therapeutic protocol (Thompson‐Brenner et al., 2019).

Despite the number of patients included in this research study, our results should be considered to have several limitations. First, the included sample was predominantly composed of AN patients, even though no psychological differences emerged between subgroups. Second, we included only female patients, which reduces confounding factors but limits the generalization of the results. Third, the psychological evaluations were only based on self‐reported questionnaires. Future studies should include more bulimia nervosa and binge eating disorder patients and men, and they should use neuropsychological evaluations of emotional recognition and management.

In conclusion, our results show that emotional regulation, alexithymia, and dissociation could be improved with specific inpatient treatment in ED patients, despite the limits of the study. Despite significant reduction, the TAS‐20 scores remained clinically significant in patients with alexithymia features, corroborating the evidence of its nature as a personality trait. Finally, we found correlations between the changes of alexithymia symptomatology and specific ED psychopathology and changes in emotional regulation, which should be further investigated due to their relevance as it pertains to treatment outcomes.

## CONFLICT OF INTEREST

On behalf of all authors, the corresponding author states that there is no conflict of interest.

## Data Availability

The clinical data are not publicly available due to privacy and ethical restrictions.
